# ﻿Notes on species of *Talaus* Simon, 1886 (Araneae, Thomisidae) from China, with descriptions of two new species

**DOI:** 10.3897/zookeys.1190.111583

**Published:** 2024-01-29

**Authors:** Cong-zheng Li, Yan-bin Yao, Yong-hong Xiao, Xiang Xu, Ke-ke Liu

**Affiliations:** 1 College of Life Science, Jinggangshan University, Ji’an 343009, Jiangxi, China Jinggangshan University Ji’an China; 2 Jinshan College of Fujian Agriculture and Forestry University, Fuzhou 350007, Fujian, China Jinshan College of Fujian Agriculture and Forestry University Fuzhou China; 3 College of Life Science, Hunan Normal University, Changsha 410081, Hunan, China Hunan Normal University Changsha China

**Keywords:** Crab spiders, new synonym, Southeast Asia, taxonomy

## Abstract

Taxonomic notes on the *Talaus* species from China are provided. Two new species, *T.yuyang* Yao & Liu, **sp. nov.** and *T.zhangjiangkou* Yao & Liu, **sp. nov.** are described and illustrated, and a further three species are redescribed based on their genitalic characters: *T.dulongjiang* Tang, Yin, Ubick & Peng, 2008, *T.niger* Tang, Yin, Ubick & Peng, 2008, and *T.sulcus* Tang & Li, 2010. The species *T.xiphosus* Zhu & Ono, 2007 is considered a junior synonym of *T.triangulifer* Simon, 1886 based on an examination of many recently collected female and male specimens from Guangxi Province, China. Diagnoses, detailed illustrations and a map of distributional records of the six treated species of *Talaus* in China are provided.

## ﻿Introduction

The crab spider genus *Talaus* Simon, 1886 comprises 13 species, of which 12 are endemic in Southeast Asia (WSC 2023); the validity of the other species, *T.limbatus* Simon, 1895 recorded from South Africa based on a juvenile specimen (Simon 1895), was doubted by Benjamin (2020). The genus has been neglected for the past ten years and only four species are known from Yunnan, Guangxi and Hainan in southwest and south China (Li and Lin 2016).

*Talaus* is currently non-monotypic with its type species, *Talaustriangulifer* Simon, 1886, described from Sumatra, Indonesia based on a female specimen (Benjamin et al. 2008; WSC 2023). Based on the combination of morphological characters of *T.beccarii* Benjamin, 2020, *T.nanus* Thorell, 1890, *T.oblitus* O. Pickard-Cambridge, 1899, *T.opportunus* (O. Pickard-Cambridge, 1873) and *T.xiphosus* Zhu & Ono, 2007, Benjamin (2020) re-defined the genus with the following features: the male palp with a retrolateral tibial apophysis and a ventral tibial apophysis, with a dorsal tibial apophysis in some species, and a short or long spiniform embolus with a broad base; the epigyne lacks a median septum, has a short copulatory duct, and the spermathecae are rounded to irregular in shape. Furthermore, Benjamin (2020) highlighted questions about the species to be included in this genus, and suggested that *T.limbatus* from South Africa was probably misplaced, that *T.xiphosus* might be a synonym of the type species *T.triangulifer*; and that *T.elegans* Thorell, 1890 might also be a junior synonym of *T.triangulifer*. When these problems are resolved, this genus will be unambiguous in the spider family Thomisidae Sundevall, 1833.

The present paper describes six species based on ample material (including some paratypes) from China which have been collected by spider enthusiasts, our colleagues, and the authors. Two new *Talaus* species are identified as undescribed and examination of these specimens reveals that *T.xiphosus* is a junior synonym of *T.triangulifer*.

## ﻿Material and methods

The new materials were collected in the bush with the malaise trap, fogging and beating method. Specimens were examined using a SZ6100 stereomicroscope. Both male and female copulatory organs were dissected and examined in 80% ethanol using an Olympus CX43 compound microscope with a KUY NICE CCD camera (Beijing Tiannuoxiang Scientific Instrument Co., Ltd, China). Epigynes were cleared with pancreatin solution (Álvarez-Padilla and Hormiga 2007).

The measurements were taken using a stereomicroscope (AxioVision SE64 Rel. 4.8.3) and are given in millimeters. The body lengths of all specimens exclude the chelicerae and spinnerets. Terminology of the male and female copulatory organs follows Benjamin (2020). Leg measurements are given as total length (femur, patella, tibia, metatarsus, tarsus). The abbreviations used in the figures are as follows:

**ALE** anterior lateral eye;

**AME** anterior median eye;

**CD** copulatory duct;

**CO** copulatory opening;

**d** dorsal;

**Em** embolus;

**EH** epigynal hood;

**FD** fertilization duct;

**MOA** median ocular area;

**p** prolateral;

**PLE** posterior lateral eye;

**PME** posterior median eye;

**r** retrolateral;

**RTA** retrolateral tibial apophysis;

**Spe** spermatheca;

**TR** tegular ridge;

**v** ventral;

**VTA** ventral tibial apophysis.

Depositories of all specimens examined are abbreviated as:

**ASM-JGSU** Animal Specimen Museum, College of Life Science, Jinggangshan University, Ji’an, China;

**CAS**California Academy of Sciences, San Francisco, USA;

**HNU**Hunan Normal University, Changsha, China.

## ﻿Taxonomy

### ﻿Family Thomisidae Sundevall, 1833

#### ﻿Genus Talaus

Taxon classificationAnimaliaAraneaeThomisidae

Simon, 1886

D2A4F311-21A3-5354-AD39-C5EBA256E015

##### Type species.

*Talaustriangulifer* Simon, 1886

##### Notes.

Before this study, the genus included 13 species (WSC 2023). One-third of these species are recorded from southern China; the validity of one species, *T.xiphosus* was doubted by Benjamin (2020) and is confirmed as a synonym in this study; therefore, only 12 *Talaus* species are restricted to Southeast Asia and the Indomalayan Realm (Bhutan, China, India, Indonesia, Myanmar, Sri Lanka, Vietnam; WSC 2023). The 13^th^ species, *T.limbatus* from South Africa, is probably misplaced.

#### ﻿ Talaus dulongjiang

Taxon classificationAnimaliaAraneaeThomisidae

Tang, Yin, Ubick & Peng, 2008

D3BB4F92-54B5-5A24-9A18-EAAA8E02EF7C

Figs 1 , 2

Talaus dulongjiang Tang et al., 2008: 63, figs 1–12.

##### Type material examined.

***Paratypes*.** 2 ♂ (DHK-2004-068): China, Yunnan. Province, Gongshan County, Dulongjiang Township, 2.3–3.3 km south of Longyuan Village along Dulongjiang, 28.00532°N, 98.32145°E, 1685 m, 2 November 2004, David Kavanaugh leg. (Tho-159, paratypes examined, HNU); 4 ♀ (Tang-04-08), Mokewang Bridge, 27.83827°N, 98.32103°E, 1455 m, 6–7 November 2004, Guo Tang leg., other data same as previous (Tho-159). Holotype not examined.

##### Diagnosis.

**Figure 1.**
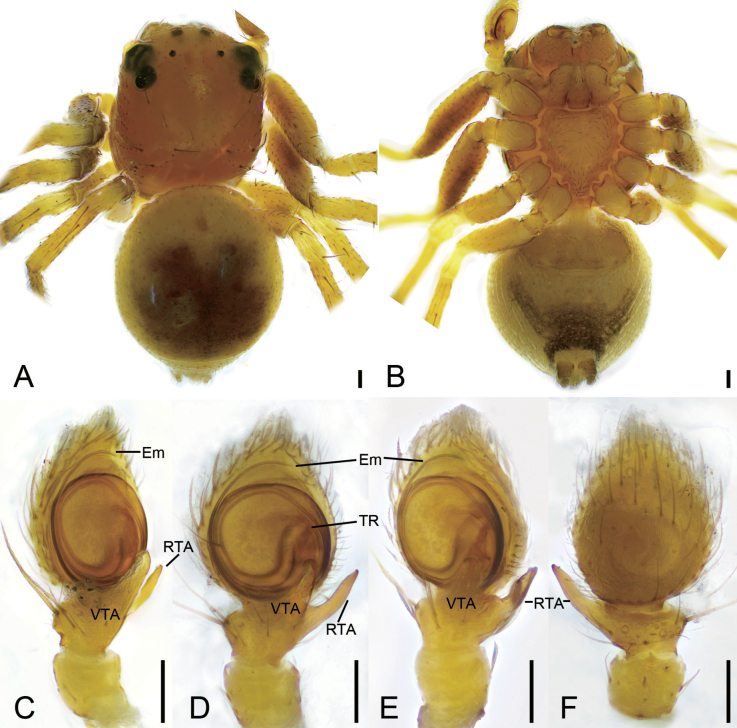
*Talausdulongjiang* Tang, Yin, Ubick & Peng, 2008, male **A** habitus, dorsal view **B** habitus, ventral view **C** palp, prolatero-ventral view **D** palp, ventral view **E** palp, ventro-retrolateral view **F** palp, dorsal view. Abbreviations: Em – embolus, RTA – retrolateral tibial apophysis, TR – tegular ridge, VTA – ventral tibial apophysis. Scale bars: 0.1 mm.

**Figure 2.**
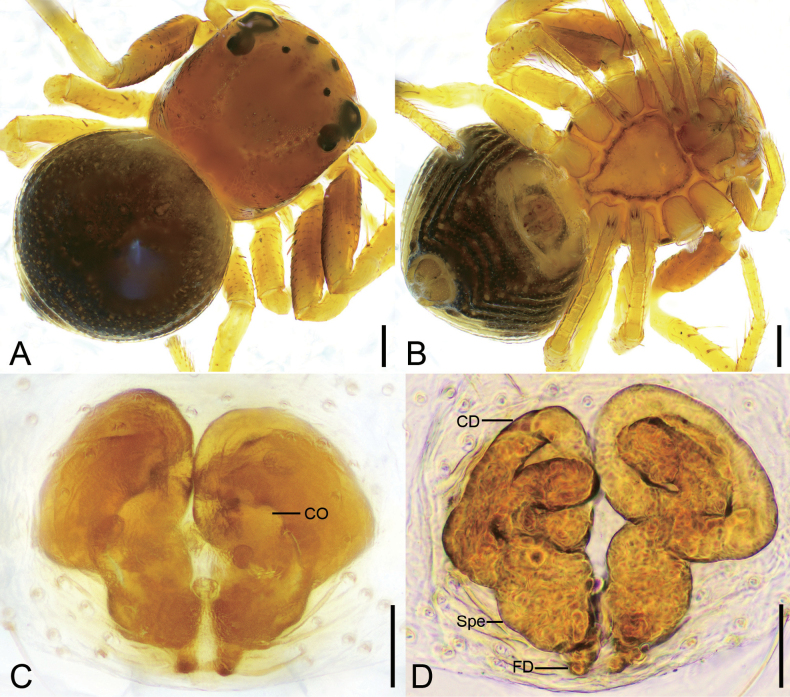
*Talausdulongjiang* Tang, Yin, Ubick & Peng, 2008, female **A** habitus, dorsal view **B** habitus, ventral view **C** epigyne, ventral view **D** epigyne, dorsal view. Abbreviations: CD – copulatory duct, CO – copulatory opening, FD – fertilization duct, Spe – spermatheca. Scale bars: 0.2 mm (**A, B**); 0.05 mm (**C, D**).

The male of this species is similar to *T.niger* Tang, Yin, Ubick & Peng, 2008 (Tang et al. 2008: 65, figs 16–18) in having the same position of the tegular ridge and the filiform embolus, but can be separated from it by the retrolateral tibial apophysis strongly bending forward (vs slightly) and the longer ventral tibial apophysis (vs relatively short) (Fig. 1C–F). The females can be easily separated from *T.niger* (Tang et al. 2008: 65, fig. 20) by the helical copulatory duct (vs S-shaped) and the slightly separated swollen spermathecae (vs clearly separated) (Fig. 2D).

##### Description.

Male habitus as in Fig. 1A, B, palp in Fig. 1C–F. ***Palp***: tibia with two apophyses, the ventral one slightly shorter than retrolateral one, but longer than tibia, with a strongly curved apex directed prolaterally; retrolateral one relatively thin, basally slightly curved antero-retrolaterally in ventral view, longer than tibia; tegular ridge long, basally arising from ~ 12 o’clock position of the tegulum; embolus filiform, spiraling nearly 3/4 coil, arising from 3 o’clock and ending at ~ 1 o’clock on tegulum. Female habitus as in Fig. 2A, B, epigyne in Fig. 2C, D. ***Epigyne***: copulatory openings relatively large, sub-antero-laterally located; copulatory ducts broad and long, looping 1.75 coils; spermathecae swollen, slightly separated by ~ 1/7 of their width; fertilization ducts located posteriorly, directed anterolaterally.

##### Distribution.

Known from Yunnan Province, China (Fig. 12).

##### Remarks.

According to Tang et al. (2008) the original material consisted of three males and seven females; two males and four females were deposited in HNU and one male and three females in CAS. However, only two males and four females (but not the holotype) were found in HNU and there are no striking markings on them: the label data match the localities of the paratypes recorded by Tang et al. (2008: 63).

#### ﻿ Talaus niger

Taxon classificationAnimaliaAraneaeThomisidae

Tang, Yin, Ubick & Peng, 2008

83943FE9-595B-5569-AC5E-3DC31173A7FB

Fig. 3

Talaus niger Tang et al., 2008c: 65, figs 13–23.

##### Type material examined.

***Paratype*.** 2 ♀ (GKJ020): CHINA, Yunnan Province, Tengchong County, Wuhe Township, Tongjiazhuang Village, Longchuanjiang River (Longjiang Bridge), along river, 24.89284°N, 98.67439°E, 1210 m, 24 May 2005, Heng-mei Yan & Ke-ji Guo leg. (Tho-202). Holotype not examined.

##### Diagnosis.

**Figure 3.**
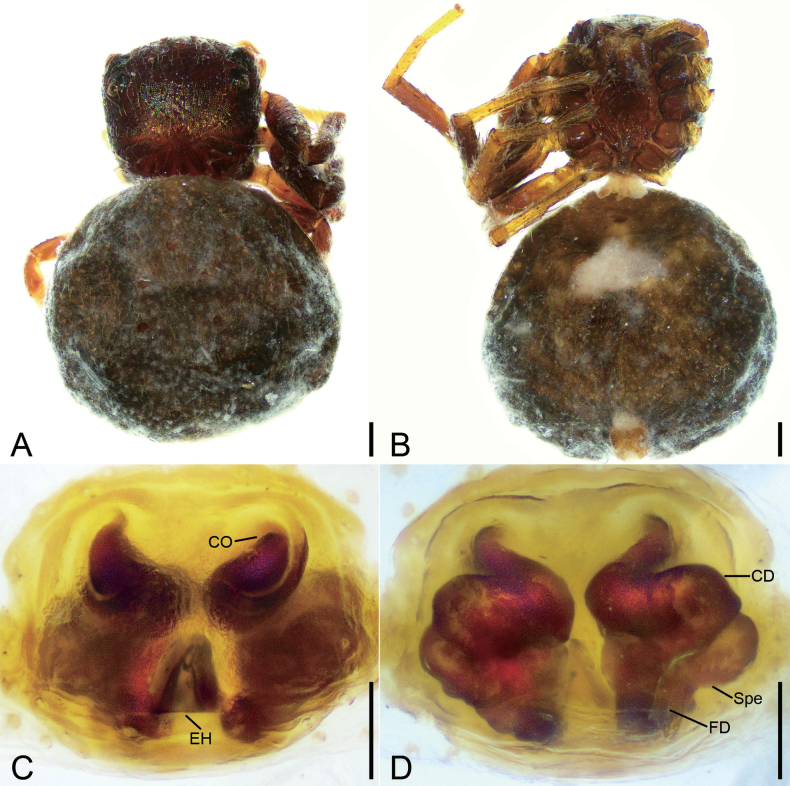
*Talausniger* Tang, Yin, Ubick & Peng, 2008, female **A** habitus, dorsal view **B** habitus, ventral view **C** epigyne, ventral view **D** epigyne, dorsal view. Abbreviations: CD – copulatory duct, CO – copulatory opening, EH – epigynal hood, FD – fertilization duct, Spe – spermatheca. Scale bars: 0.2 mm (**A, B**); 0.05 mm (**C, D**).

The female can easily be recognized by the S-shaped copulatory ducts with a swollen median part (Fig. 3D).

##### Description.

Female habitus as in Fig. 3A, B, epigyne in Fig. 3C, D. ***Epigyne***: copulatory openings relatively large, antero-medially located, with sclerotized and round margins; epigynal hood posteriorly located, thumb-like; copulatory ducts S-shaped, anterior part relative narrow, L-shaped, medial and posterior part swollen, extending transversally; spermathecae triangular, widely separated by approximately half of their width; fertilization ducts located posteriorly, directed anteriorly.

##### Distribution.

Known only from the type locality in Yunnan Province, China (Fig. 12).

##### Remarks.

According to Tang et al. (2008: 65), the original materials consisted of a male and two females, one female deposited in HNU and one female in CAS. However, two females and no male holotype were found in HNU and there are no labels designating the paratypes. Based on the locality information and the original illustration given in their paper, these specimens can be confirmed as the paratypes of *T.niger* and have been labelled as such. They are somewhat shriveled after alcohol evaporated during storage, but their epigynes still can be clearly recognized after pancreatin digestion and are now placed on labelled slides.

#### ﻿ Talaus sulcus

Taxon classificationAnimaliaAraneaeThomisidae

Tang & Li, 2010

8D5EA179-6D30-5DA9-8557-0E4E2A01D271

Figs 4 , 5

Talaus sulcus Tang & Li, 2010b: 93, f. 71A–D, 72A–F, 73A–D.

##### Other material examined.

2 ♀: China, Guangxi Zhuang Autonomous Region, Chongzuo City, Jiangzhou District, Zuozhou Town, Nongxue Village, 22°36.024'N, 107°24.93'E, 252 m, 5 September 2015, Bing Zhou, Wang Liu, Ji-he Liu, Qu Cai, Xian-feng Huang & Da Li leg. (Tho-334, HNU); 1 ♀, Guanghe Village, Pairutun, Nongzui, 22°32.556'N, 107°26.970'E, 311 m, 11 September 2015, other data same as previous (Tho-334, HNU); 1 ♀, Quxi Village, Nongqiong, 22°34.208'N, 107°25.003'E, 276 m, 31 August 2015, other data same as previous (Tho-334, HNU); 1 ♀, Longzhou County, Nonggang National Nature Reserve, Longjiang Station, Checkpoints, 22.4777°N, 106.9092°E, 204 m, 28 October 2017, Ai-lan He, Ke-ke Liu, Qu Cai, Ji-he Liu, Jin-xin Liu & Zong-guang Huang leg. (Tho-335, HNU).

##### Diagnosis.

**Figure 4.**
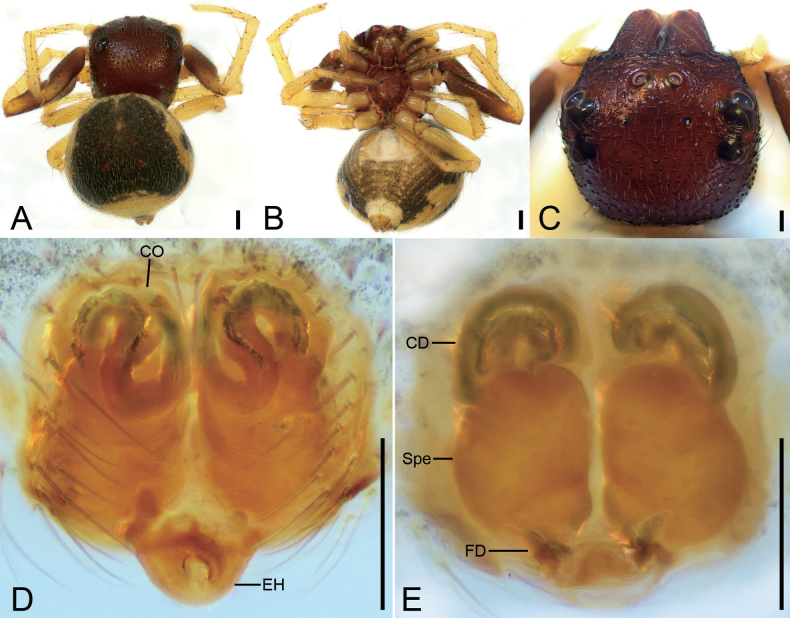
*Talaussulcus* Tang & Li, 2010, female **A** habitus, dorsal view **B** habitus, ventral view **C** prosoma, dorso-frontal view **D** epigyne, ventral view **E** epigyne, dorsal view. Abbreviations: CD – copulatory duct, CO – copulatory opening, EH – epigynal hood, FD – fertilization duct, Spe – spermatheca. Scale bars: 0.2 mm (**A, B**); 0.1 mm (**C–E**).

**Figure 5.**
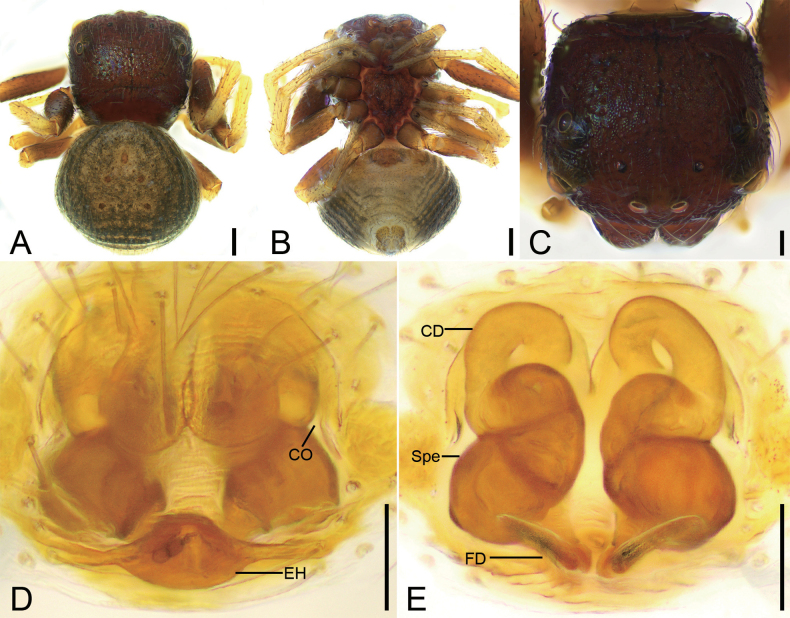
*Talaussulcus* Tang & Li, 2010, female **A** habitus, dorsal view **B** habitus, ventral view **C** prosoma, dorsal view **D** epigyne, ventral view **E** epigyne, dorsal view. Abbreviations: CD – copulatory duct, CO – copulatory opening, EH – epigynal hood, FD – fertilization duct, Spe – spermatheca. Scale bars: 0.2 mm (**A, B**); 0.1 mm (**C**); 0.05 mm (**D, E**).

Female resembles *Talaustriangulifer* (Fig. 7C, D) in having a bean-shaped spermathecae, but can be easily recognized by the copulatory openings located antero-medially and directed posteriorly (vs located antero-medially and directed anteriorly, or located latero-medially) and the S-shaped or spiral copulatory ducts (vs C-shaped) (Figs 4D, E, 5D, E).

##### Description.

Female habitus as in Figs 4A, B, 5A, B, eyes as in Figs 4C, 5C, epigyne as in Figs 4D, E, 5D, E. ***Epigyne***: copulatory openings small, antero-medially located, separated by half of width of epigynal hood; epigynal hood postero-medially located, looks like a semi-circular extrusion; copulatory ducts long, S-shaped in ventral view, C-shaped in dorsal view; spermathecae ovoid, swollen, slightly separated from each other; fertilization ducts postero-medially located, directed anterolaterally. The epigynes also include the other variations: copulatory openings small, slit-like, latero-medially located; epigynal hood postero-medially located, triangular, curved forward; copulatory ducts long, like an oval ring in dorsal view; spermathecae sac-shaped, with obvious constrictions, nearly sac-shaped, anterior slightly separated from each other anteriorly; fertilization ducts postero-medially located, touching their bases, directed anterolaterally.

##### Distribution.

Known from Yunnan (Tang and Li 2010) and Guangxi Province (present records), China (Fig. 12).

##### Remarks.

The detailed study of newly collected specimens from Guangxi revealed that they have two variants of the epigyne conformation as shown for paratype specimens from Yunnan by Tang and Li (2010). Specimens from Jiangzhou District have S-shaped (in ventral view) and C-shaped (in dorsal view) copulatory ducts and ovoid spermathecae as illustrated in Tang and Li (2010: fig. 72C, D); the specimens from Longzhou County have loop-like copulatory ducts and nearly C-shaped spermathecae similar to the illustration in Tang and Li (2010: fig. 72E, F). However, our new female specimens do not have a yellow abdomen dorsally bearing a large subtriangular spot subposteriorly, which is an acceptable color-variation proposed by Tang and Li (2010: 93).

#### ﻿ Talaus triangulifer

Taxon classificationAnimaliaAraneaeThomisidae

Simon, 1886

EE15D876-64B4-5D18-BC35-4649845C5619

Figs 6 , 7

Talaus triangulifer Simon, 1886: 172); Benjamin 2020: 414, figs 2C, H–J, 4E, 7A–B.
Talaus xiphosus Zhu & Ono, 2007: 81, figs 1–5; Benjamin 2020: 415 (“probably a synonym of T.triangulifer”). syn. nov.

##### Other material examined.

3 ♂: China, Guangxi Zhuang Autonomous Region, Chongzuo City, Longzhou County, Nonggang National Nature Reserve, Sanlian Station, Longdan, 22.53470°N, 106.83697°E, 307 m, 31 October 2017, Ai-lan He, Ke-ke Liu, Qu Cai, Ji-he Liu, Jin-xin Liu & Zong-guang Huang leg. (Tho-029, HNU); 1 ♀, 30 October 2017, other data as previous; 14 ♂ 11 ♀, Nonggang Station, Boarded-up Houses, 22.46444°N, 106.92359°E, 188 m, 28 October 2017, other data as previous; 5 ♂ 11 ♀, Core Area, 22.46415°N, 106.93238°E, 228 m, 26 October 2017, other data as previous; 4 ♂ 3 ♀, 27 October 2017, other data as previous; 6 ♂ 6 ♀, Longjiang Ligatures, 22.4770°N, 106.90921°E 204 m, 28 October 2017, other data as previous; 12 ♂ 1 ♀, 27 October 2017, other data as previous; 12 ♂ 8 ♀, Longhengtun, 22.47450°N, 106.98307°E, 270 m, 29 October 2017, other data as previous; 1 ♂ 3 ♀, 22.47166°N, 106.97051°E, 163 m, other data as previous; 1 ♂ 1 ♀, Nanning City, Wuming County, Damingshan National Nature Reserve, Sanbao Station, Chaoyang, 23°31'13.679"N, 108°23'4.560"E, 3 November 2018, 593 m, Ai-lan He, Ke-ke Liu, Hui-juan Sheng, Ji-he Liu, Jin-xin Liu & Zong-guang Huang leg.; 9 ♂ 1 ♀, Ganlan Station, 23°34'15.380"N, 108°25'16.284"E, 7 November 2018, 485 m, other data as previous; 1 ♂ 2 ♀, Shanglin County, Zhaojiang Station, 23°27'1.8"N, 108°23'32.639"E, 6 November 2018, 263 m, other data as previous; 1 ♂ 1 ♀, Jilong Station 23°26'5.279"N, 108°26'32.639"E, 591 m, 5 November 2018, other data as previous; 17 ♂ 28 ♀, Chongzuo City, Jiangzhou District, Zuozhou Town, Guanghe Village, Hecuntun, Nongyao, 22°36.318'N, 107°25.677'E, 224 m, 9 September 2015, Bing Zhou, Wang Liu, Ji-he Liu, Qu Cai, Xian-feng Huang & Da Li leg.; 8 ♂ 7 ♀, Longmitun, Nongxing, 22°34.190'N, 107°26.283'E, 272 m, 7 September 2015, other data as previous; 11 ♀, Hecuntun, Nongteng, 22°35.074'N, 107°25.430'E, 235 m, 4 September 2015, other data as previous; 1 ♂ 11 ♀, Nongdan, 22°34.054'N, 107°24.295'E, 296 m, 30 August 2015, other data as previous; 2 ♂, 12 ♀, Pairutun, 22°34.911'N, 107°25.684'E, 226 m, 3 September 2015, other data as previous; 7 ♂, 26 ♀, Nongheng, along the mountain road, 22°34.740'N, 107°24.915'E, 271 m, 29 August 2015, other data as previous; 2 ♂ 4 ♀, Duolu Town, Duobai Village, Longquantun, Nongquan, 22°32.392'N, 107°27.221'E, 145 m, 10 September 2015, other data as previous.

##### Diagnosis.

The species can be easily differentiated from other *Talaus* species by the long straight xiphoid embolus [vs curved in *T.beccarii* (Benjamin, 2020: 406, fig. 1A); short in *T.opportunus* (Benjamin 2020: 411, fig. 6A, B); flagelliform and curved in other species]. Females resemble those of *T.opportunus* (Benjamin 2020: 411, fig. 6C, D) in having a pair of question-mark-like copulatory ducts, but can be easily distinguished from it by the oval spermathecae (vs irregular in *T.opportunus*). The females are similar to those of *T.sulcus* (Tang and Li 2010: 93, fig. 72C–F) in having a semi-circular epigynal scape and the swollen spermathecae, but can be separated from it by the question-mark-like copulatory ducts (vs S-shaped or spiral in *T.sulcus*).

**Figure 6.**
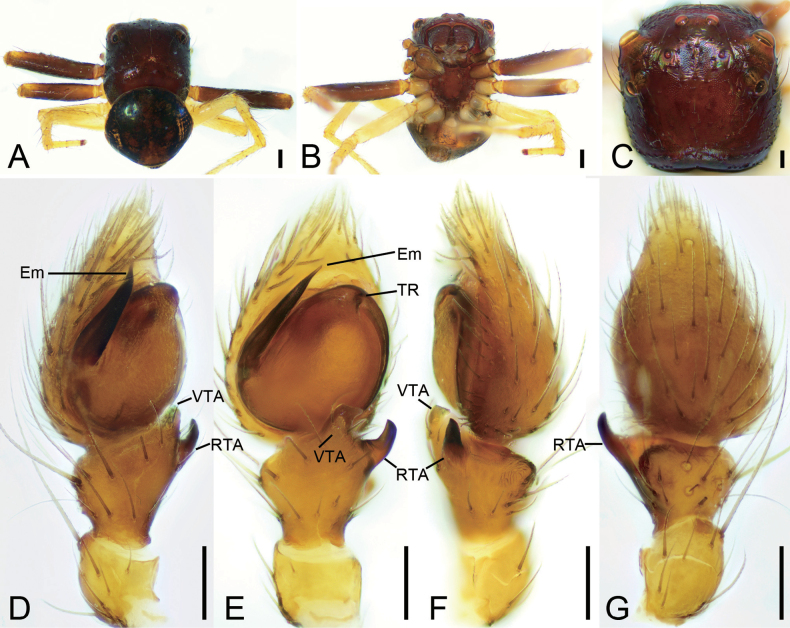
*Talaustriangulifer* Simon, 1886, male **A** habitus, dorsal view **B** habitus, ventral view **C** prosoma, dorsal view **D** palp, prolatero-ventral view **E** palp, ventral view **F** palp, retrolateral view **G** palp, dorsal view. Abbreviations: Em – embolus, RTA – retrolateral tibial apophysis, TR – tegular ridge, VTA – ventral tibial apophysis. Scale bars: 0.2 mm (**A, B**); 0.1 mm (**C–G**).

##### Description.

**Figure 7.**
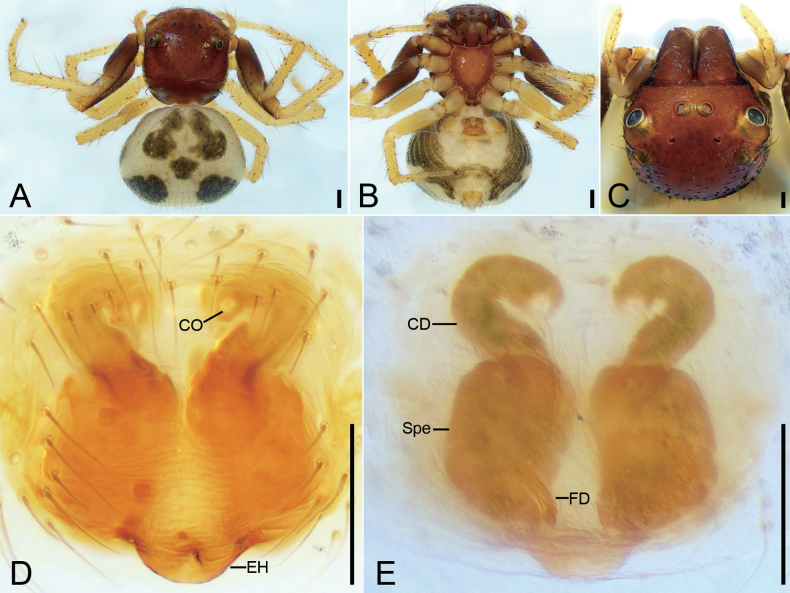
*Talaustriangulifer* Simon, 1886, female **A** habitus, dorsal view **B** habitus, ventral view **C** prosoma, dorso-frontal view **D** epigyne, ventral view **E** epigyne, dorsal view. Abbreviations: CD – copulatory duct, CO – copulatory opening, EH – epigynal hood, FD – fertilization duct, Spe – spermatheca. Scale bars: 0.2 mm (**A, B**); 0.1 mm (**C–E**).

**Male** habitus as in Fig. 6A, B, eyes as in Fig. 6C, palp as in Fig. 6D–G. ***Palp***: tibia with two apophyses: the ventral one bird-head-like in ventral view, with a sharp, narrowed apex directed retrolaterally; retrolateral one horn-like, well sclerotized, longer than ventral one; tegular ridge arising from ~ 1 o’clock position; embolus (Em) xiphoid, arising from 8 o’clock and ending at ~ 12 o’clock. Female habitus as in Fig. 7A, B, epigyne as in Fig. 7D, E. ***Epigyne***: copulatory openings small, directed backwards, separated by half width of spermathecae; epigynal hood located posteriorly, semi-circular; copulatory ducts question-mark-like; spermathecae nearly oval, swollen, anterior part nearly touching, posterior part separated by less than half of spermathecal width.

##### Distribution.

Known from Indonesia (Borneo, Sumatra; WSC 2023), Malaysia (Sabah; Benjamin 2020), and China (new records, Yunnan and Guangxi provinces; Fig. 12).

##### Remarks.

According to Zhu and Ono (2007), the original materials of *Talausxiphosus* were collected from Longzhou County, Chongzuo City in Guangxi Zhuang Autonomous Region. Of these specimens, some were collected from Chongzuo City, the others from Nanning City. Although there is 100 km from the locality of the holotype (Ningming County in Chongzuo City) to the nearest point of the locality of our specimens, and there is intraspecific variation in the abdomen color in the specimens from Zhu and Ono (2007), we did not find any noticeable differences in the male palps or in the female epigynes and therefore confirm Benjamin’s (2020) proposal that *T.xiphosus* is a junior synonym of *T.triangulifer*. The results presented here suggest that this species has a wide distribution in Southeast Asia.

#### ﻿ Talaus yuyang

Taxon classificationAnimaliaAraneaeThomisidae

Yao & Liu sp. nov.

E13AC877-4A9D-5E9C-88D6-B81B8AF3D45A

https://zoobank.org/0B1D782D-8049-473F-9399-E05EE564F269

Figs 8 , 9 , 11

##### Type material.

***Holotype*** ♂, China: Chongqing Municipality: Chengkou County, Hongjun Park, 31°56'56.89"N, 108°40'15.84"E, late October 2022, Y.Y. Zhou leg. (Tho-322, ASM-JGSU). ***Paratypes***: 1 ♂ 7 ♀, same data as holotype; 1 ♂, 1 ♀; Ledong County, Jianfengling National Natural Reserve, Mingfenggu scenic spot, 18°44'25.87"N, 108°50'47.83"E, 1–31 May 2021, Yun-hu Mo leg. (Tho-322, ASM-JGSU).

##### Etymology.

The specific name is taken from the first name of Mr Yuyang Zhou, who collected the specimens at Hongjun Park; noun in apposition.

##### Diagnosis.

The male is similar to that of *Talaussulcus* (Tang and Li 2010: 93, fig. 71B–D) in having the horn-like retrolateral tibial apophysis and the filariform embolus, but can be easily distinguished from it by the tegular ridge arising from ~ 8 o’clock position (vs 9 o’clock) and the very long finger-like ventral tibial apophysis (vs short, hump-like). The females of the new species can be easily recognized by the tunnel-like copulatory openings located anterolaterally, the very long copulatory ducts, and the spermathecae with many constrictions.

##### Description.

**Figure 8.**
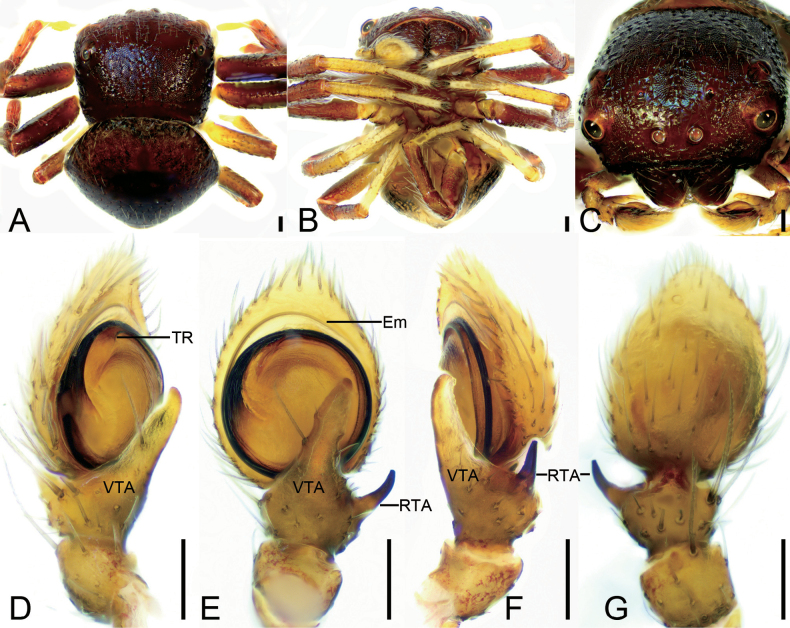
*Talausyuyang* sp. nov., male holotype **A** habitus, dorsal view **B** habitus, ventral view **C** prosoma, dorso-frontal view **D** palp, prolatero-ventral view **E** palp, ventral view **F** palp, retrolateral view **G** palp, dorsal view. Abbreviations: Em – embolus, RTA – retrolateral tibial apophysis, TR – tegular ridge, VTA – ventral tibial apophysis. Scale bars: 0.1 mm.

**Male** (holotype). ***Habitus*** (Figs 8A, B, 11). Total length 1.68. ***Carapace*** (Fig. 8A) red brown, length 0.71, width 1.0, with densely short setae. ***Eyes*** (Fig. 8C) diameters and interdistances: AME 0.05, ALE 0.11, PME 0.05, PLE 0.07; AME–AME 0.13, ALE−AME 0.26, PME–PME 0.27, PLE−PME 0.26, AME−PME 0.14, AME−PLE 0.42, ALE−ALE 0.76, PLE−PLE 0.79, ALE−PLE 0.18. MOA 0.24 long, front width 0.24, back width 0.38. Chelicerae red brown, straight, robust, without retromarginal or promarginal teeth. Endites yellow brown, longer than wide. Labium yellow brown, longer than wide. Sternum red brown, longer than wide. Legs red brown except yellow metatarsi and tarsi (Fig. 8A, B); measurements: I 2.33 (0.71, 0.29, 0.51, 0.5, 0.32); II 2.41 (0.71, 0.29, 0.54, 0.53, 0.34); III 1.63 (0.48, 0.23, 0.35, 0.31, 0.26); IV 1.62 (0.49, 0.24, 0.38, 0.29, 0.22); spination: I Pa: d1; Ti: d2, p2, r2, v2; Mt: d4, p2, r2, v4; II Pa: d1; Ti: d2, p2, r2, v2; Mt: d4, p1, r2, v4; III Pa: d1; Ti: d2, p1; Mt: d2; IV: Pa: d1; Ti: r1; Mt: d1, p1, r1. ***Abdomen*** (Fig. 8A, B) 0.98 long, 1.12 wide, ovoid, black brown; venter yellow.

***Palp*** (Fig. 8D−G). Ventral tibial apophysis digitiform, longer than tibia, apex slightly curved retrolaterally. Retrolateral tibial apophysis slightly shorter than tibia, horn-like, slightly curved in ventral view. Tegular ridge located at ~ 10 o’clock. Embolus filiform, arising from 9 o’clock and ending at ~ 3 o’clock, spiraling 1.5 coils.

**Figure 9.**
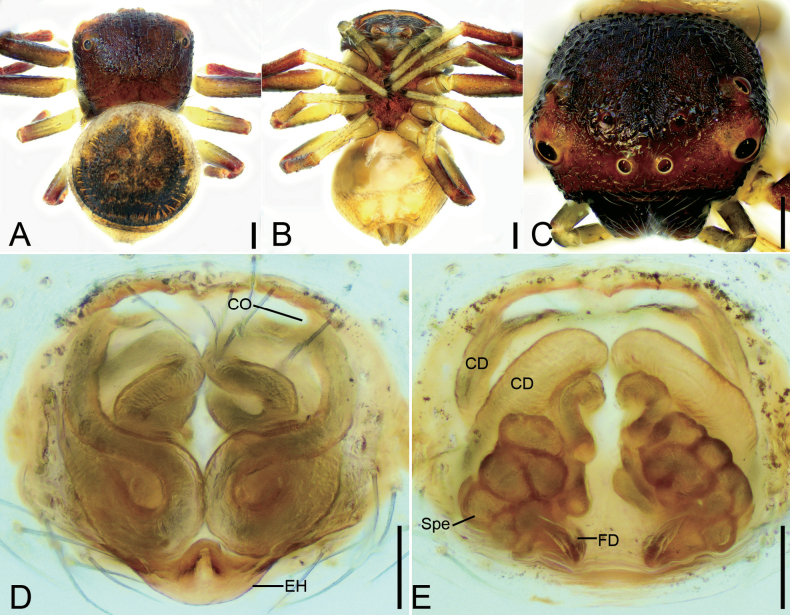
*Talausyuyang* sp. nov., female paratype **A** habitus, dorsal view **B** same, ventral view **C** prosoma, dorso-frontal view **D** epigyne, ventral view **E** same, dorsal view. Abbreviations: CD – copulatory duct, CO – copulatory opening, EH – epigynal hood, FD – fertilization duct, Spe – spermatheca. Scale bars: 0.2 mm (**A–C**); 0.05 mm (**D, E**).

**Female** (paratype). ***Habitus*** (Fig. 9A, B). As in male except as follows. Total length 1.84. ***Carapace*** (Fig. 9A) broadly square, length 0.83, width 0.95, with densely short setae. ***Eye*** (Fig. 9C) diameters and interdistances: AME 0.05, ALE 0.09, PME 0.05, PLE 0.07; AME–AME 0.11, ALE−AME 0.26, PME–PME 0.24, PLE−PME 0.26, AME−PME 0.16, AME−PLE 0.41, ALE−ALE 0.72, PLE−PLE 0.71, ALE−PLE 0.19. MOA 0.24 long, front width 0.22, back width 0.33. Chelicerae yellow, with abundant thick setae on frontal surface. ***Legs measurements***: I 1.98 (0.65, 0.27, 0.41, 0.36, 0.29); II 2.09 (0.71, 0.31, 0.45, 0.34, 0.28); III 1.32 (0.33, 0.24, 0.28, 0.23, 0.24); IV 1.51 (0.48, 0.22, 0.35, 0.27, 0.19); spination: I Fe: d2, p2; Ti: p2, v2; Mt: d4, p2, v2; II Ti: p2; Mt: d4, p2, v2; III Fe: d1; Pa: d1; Mt: d2, p2; IV: Fe: d1; Pa: d1; Mt: d2, p1. ***Abdomen*** (Fig. 9A, B) ovoid, 1.01 long, 1.05 wide, yellow to black brown, with yellow margin dorsally; venter yellow.

***Epigyne*** (Fig. 9D, E). Copulatory openings located at antero-lateral part of atrium, transversal, tunnel-shaped. Copulatory ducts very long, convoluted, double S-shaped in ventral view, splay in dorsal view. Spermathecae sac-shaped, with many constrictions its surface. Fertilization ducts blade-like, directed anterolaterally.

##### Distribution.

Known only from the Chongqing Municipality and Hainan Province of China (Fig. 12).

#### ﻿ Talaus zhangjiangkou

Taxon classificationAnimaliaAraneaeThomisidae

Yao & Liu sp. nov.

20D5D046-05A6-5B0D-B06D-36F39D58C638

https://zoobank.org/6945DACB-01F3-472D-9459-95C013CD83D1

Fig. 10

##### Type material.

***Holotype*** ♂, China: Fujian Province: Zhangzhou City, Yunxiao County, Dongxia Town, Fujian Zhangjiangkou National Mangrove Nature Reserve, 23°55'38.08"N, 117°24'52.91"E, 4 March 2023, H.T. Song, Z.H. Qi, R.X. Su, and B. Ding leg. (Tho-347, ASM-JGSU).

##### Etymology.

The specific name is taken from the type locality; noun in apposition.

##### Diagnosis.

**Figure 10.**
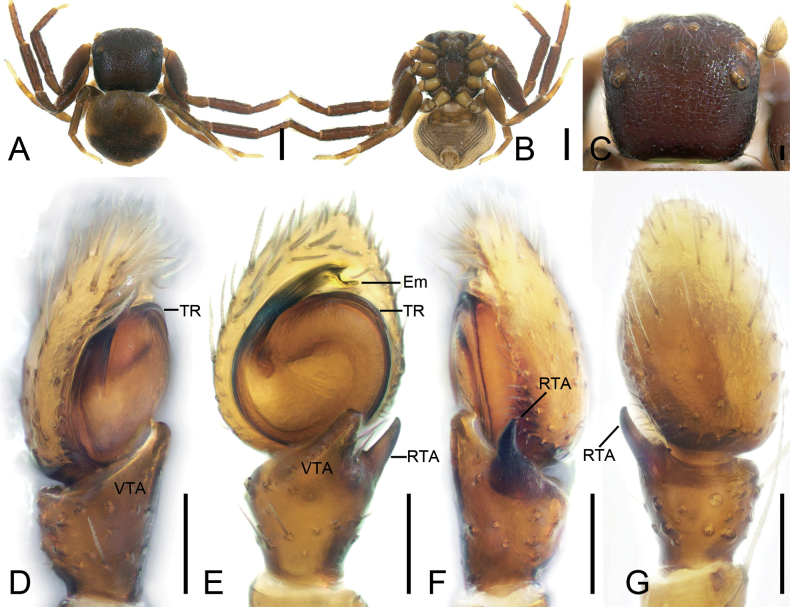
*Talauszhangjiangkou* sp. nov., male holotype **A** habitus, dorsal view **B** habitus, ventral view **C** prosoma, dorsal view **D** palp, prolatero-ventral view **E** palp, ventral view **F** palp, retrolateral view **G** palp, dorsal view. Abbreviations: Em – embolus, RTA – retrolateral tibial apophysis, TR – tegular ridge, VTA – ventral tibial apophysis. Scale bars: 0.1 mm (**A, B**); 0.1 mm (**C–G**).

This new species is similar to that of *Talaustriangulifer* (Benjamin 2020: 414, figs 2I, J, 7A, B) in having the longer, broad-based embolus and the configuration of the tibial apophyses, but can be easily distinguished from it by the retrolateral tibial apophysis with a distinctly curved tip directed mostly dorsally as seen in retrolateral view (vs directed mostly ventrally) and the embolus with a furcate tip (vs pointed) in ventral view (Fig. 10E–G).

##### Description.

***Habitus*** (Fig. 10A, B). Total length 2.24. ***Carapace*** (Fig. 10A) red brown, length 1.07, width 1.14, with densely short setae. ***Eyes*** (Fig. 10C) diameters and interdistances: AME 0.07, ALE 0.12, PME 0.05, PLE 0.08; AME–AME 0.12, ALE−AME 0.31, PME–PME 0.32, PLE−PME 0.3, AME−PME 0.17, AME−PLE 0.5, ALE−ALE 0.88, PLE−PLE 0.88, ALE−PLE 0.21. MOA 0.29 long, front width 0.25, back width 0.41. Chelicerae red brown, straight, robust, without retromarginal or promarginal teeth. Endites yellow brown, longer than wide. Labium yellow brown, longer than wide. Sternum red brown, longer than wide, with long dense setae. Legs red brown except yellow metatarsi and tarsi (Fig. 10A, B); measurements: I 3.24 (1, 0.42, 0.77, 0.61, 0.44); II 3.49 (1.04, 0.48, 0.81, 0.7, 0.46); III 2.23 (0.68, 0.32, 0.54, 0.38, 0.31); IV 2.08 (0.67, 0.3, 0.48, 0.37, 0.26); spination: I Pa: d1, p1; Ti: d3, p2, r3, v2; Mt: d3, p1, r1, v4; II Ti: d3, p3, r3, v2; Mt: d3, p2, r1, v3; III Pa: d1; Ti: d1, r1, v1; Mt: d2, p1, r1, v1; IV: Ti: d1, r1; Mt: d1, p1, r1, v1. ***Abdomen*** (Fig. 10A, B) 1.17 long, 1.37 wide, ovoid, with a round and a fan-shaped black-brown spots dorsally; venter yellow, with black sloping stripes.

***Palp*** (Fig. 10D−G). Ventral tibial apophysis digitiform, shorter than tibia, apex slightly curved retrolaterally. Retrolateral tibial apophysis nearly as long as tibia, horn-like, slightly curved in retrolateral view, directed mostly dorsally. Tegular ridge arising from ~ 12 o’clock. Embolus stout, with broad base, apically furcate, arising from 9 o’clock and ending at ~ 1 o’clock.

**Female.** Unknown.

##### Distribution.

Known only from the Fujian Province of China (Fig. 12).

**Figure 11.**
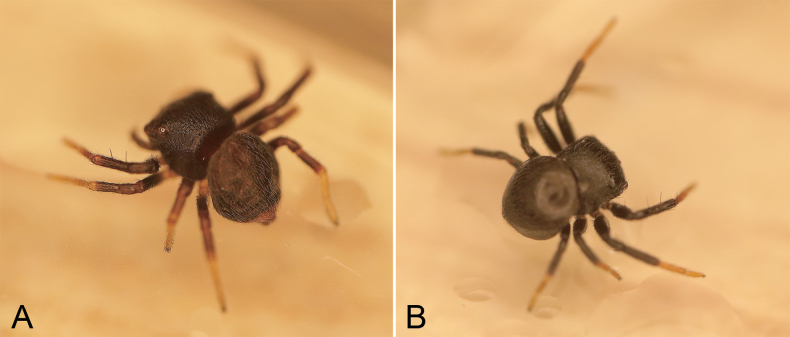
Photographs of live specimen from China **A, B***Talausyuyang* sp. nov., male.

**Figure 12.**
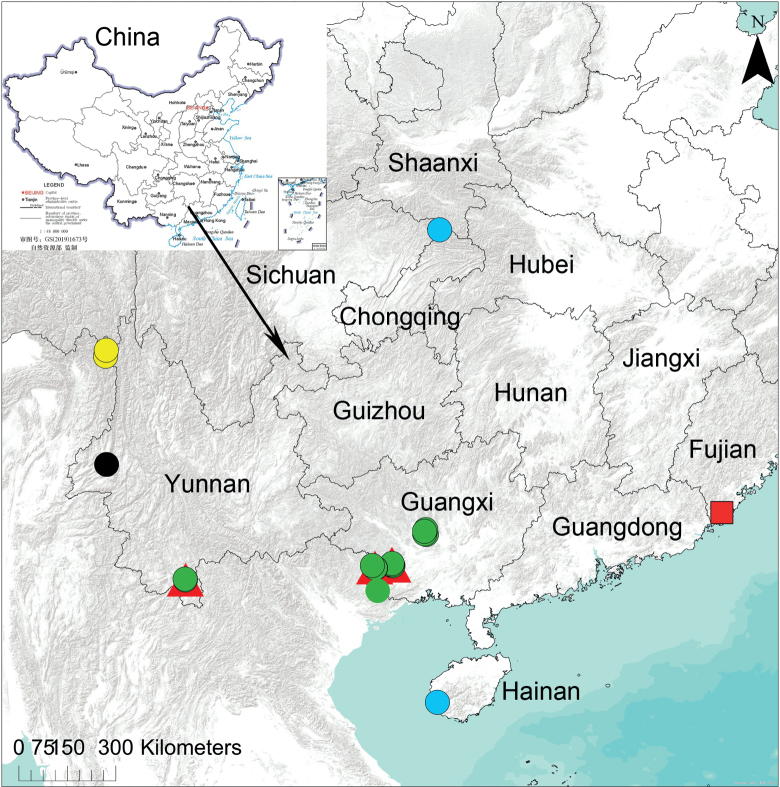
Distributional records of *Talausdulongjiang* Tang, Yin, Ubick & Peng, 2008 (yellow circles), *T.niger* Tang, Yin, Ubick & Peng, 2008 (black circle), *T.sulcus* Tang & Li, 2010 (red triangles), *T.triangulifer* Simon, 1886 (green circles), *T.yuyang* sp. nov. (blue circles) and *T.zhangjiangkou* sp. nov. (red square) from China.

## Supplementary Material

XML Treatment for Talaus

XML Treatment for Talaus dulongjiang

XML Treatment for Talaus niger

XML Treatment for Talaus sulcus

XML Treatment for Talaus triangulifer

XML Treatment for Talaus yuyang

XML Treatment for Talaus zhangjiangkou

## ﻿Additional information

### Author ORCIDs

Cong-zheng Li https://orcid.org/0000-0003-3849-8433

Yan-bin Yao https://orcid.org/0000-0002-2560-9299

Yong-hong Xiao https://orcid.org/0000-0002-8074-9366

Xiang Xu https://orcid.org/0000-0001-9485-5373

Ke-ke Liu https://orcid.org/0000-0001-7822-3667

### Data availability

All of the data that support the findings of this study are available in the main text.

## References

[B1] Álvarez-Padilla F Hormiga G (2007) A protocol for digesting internal soft tissues and mounting spiders for scanning electron microscopy. The Journal of Arachnology 35(3): 538–542. 10.1636/Sh06-55.1

[B2] Benjamin SP (2020) Distributional and taxonomic notes on the crab spider genus *Talaus* Simon, 1886 with description of a new species (Araneae: Thomisidae). Zootaxa 4858(3): 405–416. 10.11646/zootaxa.4858.3.6 33056222

[B3] Benjamin SP Dimitrov D Hormiga G Gillespie RG (2008) Family ties: molecular phylogeny of crab spiders (Araneae: Thomisidae). Cladistics 24(5): 708–722. 10.1111/j.1096-0031.2008.00202.x

[B4] Li SQ Lin YC (2016) Species Catalogue of China (Vol. 2). Animals. Invertebrates (1). Arachnida: Araneae. Science Press, Beijing, 549 pp.

[B5] Simon E (1895) Descriptions d’arachnides nouveaux de la famille des Thomisidae. Annales de la Société Entomologique de Belgique 39: 432–443.

[B6] Tang G Li SQ (2010) Crab spiders from Xishuangbanna, Yunnan Province, China (Araneae, Thomisidae). Zootaxa 2703(1): 1–105. 10.11646/zootaxa.2703.1.1

[B7] Tang G Yin CM Ubick D Peng XJ (2008) Two new species of the crab spider genus *Talaus* (Araneae: Thomisidae) from Yunnan Province, China. Zootaxa 1815(1): 62–68. 10.11646/zootaxa.1815.1.6

[B8] WSC (2023) World Spider Catalog. Natural History Museum Bern. Version 24. https://wsc.nmbe.ch/ [Accessed 22 April 2023]

[B9] Zhu MS Ono H (2007) New record of the spider genus *Talaus* from south China, with description of a new species (Araneae: Thomisidae). Acta Arachnologica 56(2): 81–83. 10.2476/asjaa.56.81

